# Small bowel obstruction secondary to migration of a fragment of lithobezoar: a case report

**DOI:** 10.1186/1757-1626-2-9155

**Published:** 2009-12-07

**Authors:** Mekki Medani, Eddie Myers, Bryan Kenny, David Waldron

**Affiliations:** 1 Department of Surgery & Radiology, Mid-Western Regional Hospital, Limerick, Ireland

## Abstract

**Introduction:**

Small bowel obstruction is a common world-wide condition that has a range of etiological factors. The management is largely dependent on the cause of the obstruction. Small bowel obstruction caused by foreign body ingestion is rare; many items have been reported as responsible, but there are no reports implicating polyurethane foam.

**Case presentation:**

We report the case of a 44-year-old Irish male who presented following ingestion of polyurethane foam. He was asymptomatic on presentation but developed a small bowel obstruction shortly thereafter.

**Conclusion:**

Patients presenting following ingestion of polyurethane foam should be scheduled for elective laparotomy, gastrotomy, and retrieval of the cast on the next available theatre list - given that they are suitable for surgery.

## Case Presentation

**Figure 1:**
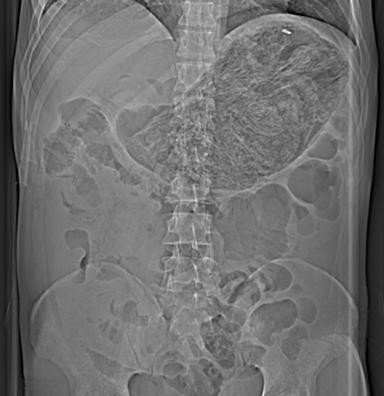
**Intra-gastric cast**.

We report the case of a 44-year-old gentleman with a background history of multiple laparotomies for deliberate self harm who presented six weeks following ingesting of polyurethane self-expanding foam. On admission, the patient had no signs or symptoms of small bowel obstruction. A plain film of his abdomen confirmed the presence of an intra-gastric cast of self-expanding foam (Figure 1).

**Figure 2:**
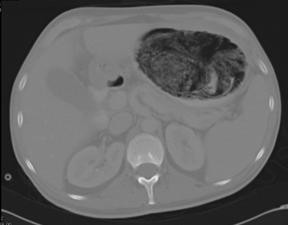
**CT abdomen showing the large intra-gastric lithobezoar**.

A planned elective operation for laparotomy and retrieval of the cast was organized. In the interim, he went on to develop a small bowel obstruction. A CT scan of his abdomen confirmed the small bowel obstruction to be secondary to migration of a fragment of foam (Figure 2).

**Figure 3:**
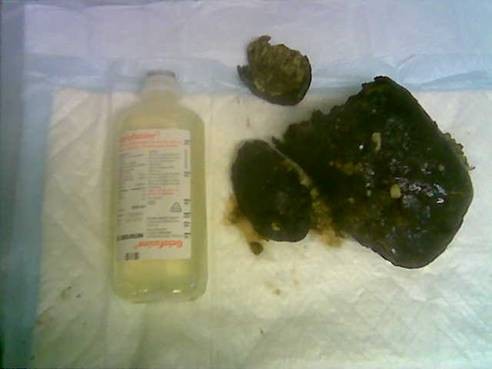
**Cast following extraction**.

The obstruction settled with conservative management. Thereafter, he underwent a laparotomy, gastrotomy, and removal of the cast on an elective basis (Figure 3). He made an uneventful post-operative recovery.

## Discussion

Bowel obstruction was, until the late 19^th ^and early 20^th ^century, a concern of the physician rather than the surgeon. The value of surgical intervention was only recognized after the advent of anaesthesia and antisepsis.

In the developed world, intra-abdominal adhesions are recognized as the leading cause of small bowel obstruction, accounting for over 60% of all cases. Small bowel obstruction secondary to foreign bodies remains a rare cause.

Small bowel obstruction (SBO) secondary to foreign bodies (FB) is rare; it represents less than 6% of all SBO cases [1], and causes serious morbidity in less than one percent of all patients [2]. It is, however, commoner in certain cohorts, including children, and patients with alcoholism or psychosis [3]. Bezoar is the term used to describe the accumulation of undigested foreign bodies or food material in the gastrointestinal tract forming a conglomeration. The constituents of a bezoar generally dictate their nomenclature; e.g. phytobezoar (fibers or seeds of vegetables and fruits); trichobezoar (hair); lactobezoar (remnants of milk) and lithobezoar (rock or similar substances). Although they can be found in any part of the gastrointestinal system, the stomach is the most common site for bezoars [4].

Generally, bezoars remain in the stomach; however, they will occasionally pass to the small bowel [5]. Unlike in this case, initial radiographic imaging is frequently non-diagnostic with regards to the etiology of the obstruction, as the bezoars are not usually visible on plain films.

Other reported FB causing SBO include mesh plugs following open hernia repair, retained surgical gauze, magnetic toys, endoscopy capsules, retained PEG tube internal bumpers, gastric bands, and many others.

The signs and symptoms of FB SBO are the same as those of SBO secondary to any other cause, however a history of FB ingestion is usually evident; otherwise, a high index of suspicion is necessary, especially in children and patients with psychiatric illnesses.

In the management of FB ingestion, there are recommendations that are in place, recommended by a panel of experts [6]. These suggest that any foreign body that has not passed the stomach in three to four weeks should be removed endoscopically; which would not have been possible in this case. It also suggests that blunt objects beyond the stomach that remain in the same location for more than one week should be considered for surgical removal; and FB causing fever, vomiting, abdominal pain, or significant symptoms should be considered for emergency removal [6].

## Conclusion

Patients presenting following ingestion of self-expanding foam should be scheduled for surgical retrieval on the next available elective list, as they are at risk of developing small bowel obstruction, due to the fragmentation of the lithobezoar.

## Consent

Written informed consent was obtained from the patient for publication of this case report and accompanying images.

A copy of the written consent is available for review by the Editor-in-Chief of this journal.

## Competing interests

The authors declare that they have no competing interests.

## Authors' contributions

MM collected the patient information, performed the literature review, and was the major contributor in writing the manuscript. EM edited the manuscript and provided critical analysis of the body. BK interpreted the imaging and contributed to the image selection in the manuscript.

All authors have read an approved this final manuscript.
